# Circular RNAs in the Central Nervous System

**DOI:** 10.3389/fmolb.2021.629593

**Published:** 2021-03-19

**Authors:** Meng-Lan Li, Wen Wang, Zi-Bing Jin

**Affiliations:** Beijing Institute of Ophthalmology, Beijing Tongren Eye Center, Beijing Tongren Hospital, Capital Medical University, Beijing Ophthalmology & Visual Sciences Key Laboratory, Beijing, China

**Keywords:** circular RNA, review (article), function, disease, central nervous system

## Abstract

Circular RNAs (circRNAs) are endogenous single-stranded RNAs characterized by covalently closed loop structures with neither 5′ to 3′ polarity nor poly(A) tails. They are generated most commonly from back-splicing of protein-coding exons. CircRNAs have a tissue-specific distribution and are evolutionarily conserved, and many circRNAs play important biological functions by combining with microRNAs and proteins to regulate protein functions and their own translation. Numerous studies have shown that circRNAs are enriched in the central nervous system (CNS) and play an important role in the development and maintenance of homeostasis. Correspondingly, they also play an important role in the occurrence and progression of CNS diseases. In this review, we highlight the current state of circRNA biogenesis, properties, function and the crucial roles they play in the CNS.

## Introduction

For many years, although proven to be present, circular RNAs (circRNAs) were overlooked as byproducts of splicing errors (Sanger et al., 1976; Barrett and Salzman, 2016). However, major recent studies have discovered that circRNAs have essential functions and play a novel regulatory role, especially in the nervous system (Memczak et al., 2013), prompting interest from an increasing number of investigators from various fields. Unlike messenger RNAs (mRNAs), circRNAs are mostly derived from different regions of gene loci in eukaryotes through a non-canonical splicing process called “back-splicing.” During back-splicing, the downstream 5′ splice site is covalently bonded to an upstream 3′ splice site in a reversed orientation. Due to the lack of 5′–3′ polarity and a polyadenylated tail, circRNAs are much more insusceptible than linear RNAs to degradation by exonuclease RNase R. CircRNAs exert their action mainly by acting as a miRNA sponge and functioning through a competing endogenous RNA (ceRNA) mechanism (Hansen et al., 2013; Memczak et al., 2013), as well as acting as protein sponges or translating proteins (Legnini et al., 2017). Recent genome-wide profiling of circRNAs has shown that numerous circRNAs are widely and dynamically expressed in the nervous system. Furthermore, their expression is dramatically increased in the brain during the aging of multiple organisms (Knupp and Miura, 2018). In addition, circRNAs participate in many processes of neurological diseases. For instance, circ-TTBK2 and circPCMTD1 act as sponges of miR-224-5p to promote glioma progression (Zheng et al., 2017, 2019). As an extension of the brain and a part of the central nervous system (CNS), the retina is another option for investigators to study enigmas of circRNAs. It has been demonstrated that circRNAs are abundant in the retina and play a role in biogenesis and various biological functions (Wang et al., 2018a; Chen et al., 2020c).

## Biogenesis, Properties, and Functions of circRNAs

### Biogenesis

**FIGURE 1:**
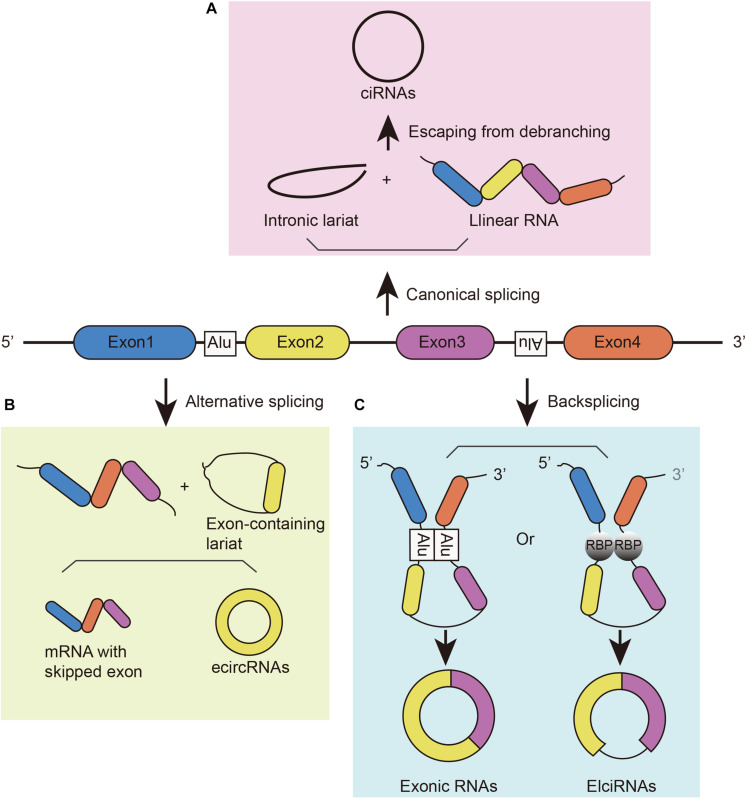
The biogenesis of circRNAs. **(A)** CiRNAs are generated from intronic lariat precursors that escape from the debranching step of canonical linear splicing. **(B)** EcircRNAs can be generated from exon-containing lariats created by an exon-skipping event during linear splicing. **(C)** CircRNAs can be generated from back-splicing mediated by inverted repeat elements and *trans*-acting RNA binding proteins.

Circular RNAs can be generated from different gene loci, such as coding and non-coding exons, introns, both exons and introns, or antisense or intergenic sequences. Due to their different origins, they have different names, such as exonic circRNA (or ecircRNA), intronic circRNA (or ciRNA), and exon–intron circRNA (or ElciRNA) (Figure 1; Zhang et al., 2013; Guo et al., 2014; Li et al., 2015). These circRNAs can be generated in different manners. (1) They can be generated from intronic lariat precursors that escape from the debranching step of canonical linear splicing (Figure 1A; Zhang et al., 2013). A pre-mRNA can generate linear RNA, lariat introns, Y-structure introns from trans-splicing, and circular exons through exon skipping (Suzuki et al., 2006). In canonical splicing, a lariat intron is generated after the splicing of the linear pre-mRNA and isolated from the ultimate mRNA product. This excised lariat undergoes internal back-splicing. Then, lariats that escape from debranching can lead to the formation of ciRNAs (Zhang et al., 2013; Eger et al., 2018). (2) Lariat formation during exon skipping (Figure 1B; Kelly et al., 2015; Petkovic and Muller, 2015). During alternative splicing, an exon-skipping event occurs and creates an exon-containing lariat formation, intronic lariat formation and mRNA with skipped exons (Zaphiropoulos, 1997; Barrett et al., 2015). The exon-containing lariat creates ecircRNAs when undergoing internal back-splicing (Kelly et al., 2015; Petkovic and Muller, 2015). However, not all of the exon-containing lariats will generate circular RNA despite the correlation between exon-skipping and circular RNA formation (Barrett et al., 2015; Kelly et al., 2015). And the smaller skipped exons are less prone to circularize than those in large size (Barrett et al., 2015). (3) Looped by base pairing between inverted repeat elements (such as Alu elements) (Jeck et al., 2013; Ivanov et al., 2015) or RNA-binding proteins (RBPs) (Figure 1C; Errichelli et al., 2017). Looping of the intron sequences flanking the downstream splice-donor site and the upstream splice-acceptor site brings these sites into close proximity (Ivanov et al., 2015). RBPs can bind two flanking introns together to promote the formation of circular structures; then, introns will be removed to form junction sites. For instance, muscleblind protein (MBL) provokes circMbl biogenesis in the form of RBPs, resulting in competition between circMbl and its linear transcript (Ashwal-Fluss et al., 2014). Similarly, the immune factors NF90/NF110 also promote circRNA production in the nucleus as RBPs (Li et al., 2017). In addition, some cis-regulatory elements and trans-acting factors participate in back-splicing, such as the spliceosome assembly (Zhang et al., 2016). Although studies have illustrated these mechanisms of circRNA biogenesis, this process has not been fully elucidated.

### Properties

As circular structure, circRNAs have some common characteristics. First, circRNAs are prevalent across species and evolutionarily conserved. They can be detected in many species, from plants to animals and from *Caenorhabditis elegans* to humans (Memczak et al., 2013; Sun et al., 2019). With a strict definition, investigators found that 4,522 of 15,849 mouse circRNAs were conserved in humans (Rybak-Wolf et al., 2015). For example, the circTulp4 isoform is derived from homologous exons in humans and mice (Rybak-Wolf et al., 2015). Second, circRNAs have tissue/developmental-stage-specific expression. For instance, circCdr1as is abundantly expressed in the nervous system, whereas it is barely detected in non-neuronal tissues (Memczak et al., 2013; Chen et al., 2020b). In addition, circRNAs are dynamically expressed during development. In the mouse hippocampus, circRNAs derived from protein coding gene loci with synapse-related functions were observed to be upregulated from E18 to P30 [You et al. (2015) profiled four stages: embryonic (E18), early postnatal (P1), the beginning of synapse formation (P10), and late postnatal (P30)]. In contrast, those produced from gene loci without any function were downregulated. Third, circRNAs are much more resistant than linear RNAs. Due to the closed loop structure, circRNAs are stable and can resist degradation from RNase R, which indicates why circRNAs can accumulate in cells for a long time (You et al., 2015). Fourth, circRNAs have incredible diversity. Different circRNAs can consist of one or more exons or even no exons (intronic circRNA), but they principally contain 2–5 exons (Rybak-Wolf et al., 2015). The length of circRNAs ranges from 100 bp to 4 kb (Salzman et al., 2013).

### Biological Function

**FIGURE 2:**
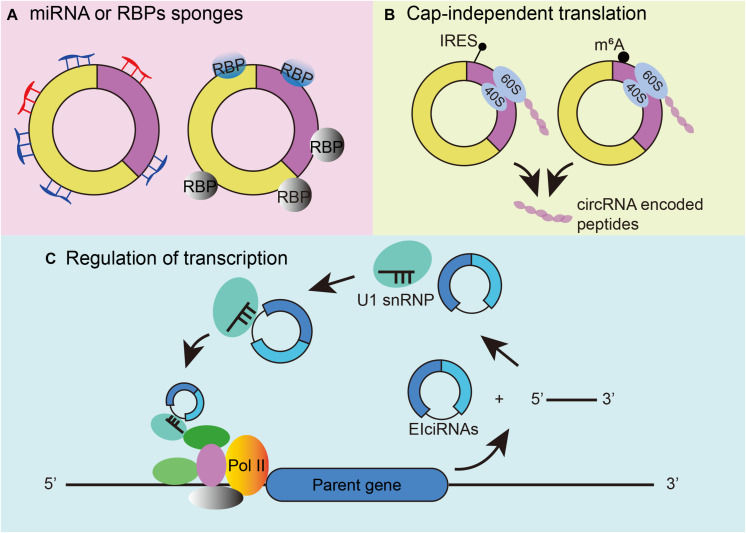
Mechanisms of circRNA functions. **(A)** CircRNAs can function as microRNA and RBP sponges. **(B)** CircRNA cap-independent translation mechanism: IRES-driven circRNA translation (left) and m6A-driven circRNA translation (right). **(C)** Regulation of transcription initiation by EIciRNAs.

As a novel type of RNA, circRNAs have been proven to play various roles in biological processes. First, individual circRNAs have been posited to function as miRNA or RNA-binding protein sponges (Figure 2A). A famous circRNA, ciRS-7, also known as circCdr1as, has more than 70 putative binding sites for miR-7, allowing multiple interactions. Knockdown of circCdr1as decreased the expression of miR-7 target genes, whereas knockout of circCdr1as downregulated miR-7 (Hansen et al., 2013; Memczak et al., 2013; Piwecka et al., 2017). Regarding the combination of RBP, the tumor suppressor gene Foxo3 can produce circFoxo3, and circFoxo3 regulates cell cycle progression by binding to cell division protein kinase 2 (CDK2) and cyclin-dependent kinase inhibitor 1 (p21) (Diallo et al., 2019). Second, circRNA can be translated in cap-independent manners. Under stress conditions, circRNA can use its internal ribosome entry site (IRES) to recruit ribosomes to an internal position of circRNA. For example, circMbl shares the same start codon as the linear mRNA and can be found with increasing IRES activity. The proteins detected by mass spectrometry also provide important evidence (Pamudurti et al., 2017) (Figure 2B left). The second mechanism for circRNA translation is the recruitment of eukaryotic initiation factor 3 (eIF3) by methylated adenosine residues in the form of N6-methyladenosines (m6A) in the 5′untranslated region (5′UTR) for translation into small polypeptides (Diallo et al., 2019; Zhou et al., 2021) (Figure 2B right). Third, circRNA can participate in transcriptional regulation by interacting with RNA polymerase II (RNA pol II) and other snRNP partners. Experiments have shown that circEIF3J and circPAIP2 can regulate the transcription of their parental genes through this mechanism (Li et al., 2015; Figure 2C).

## Distribution, Developmental-Stage-Specific Expression Profile, and Age-Related Accumulation of circRNAs in the Nervous System

### Distribution

**FIGURE 3:**
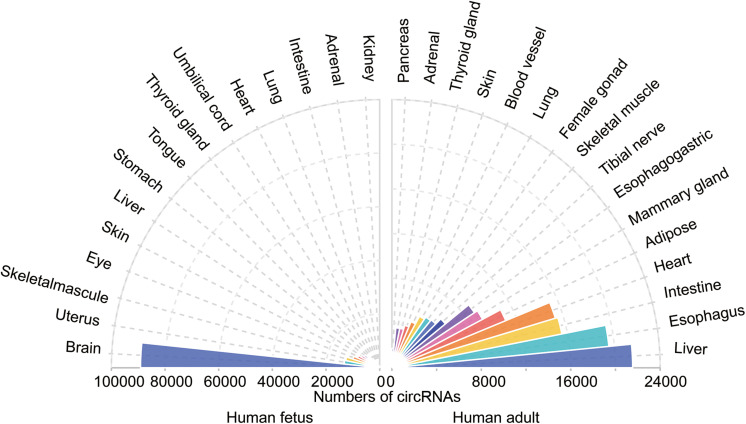
The tissue-specific circRNAs expressed in human tissues. The colorful triangles represent the numbers of circRNAs detected in different human tissues (left, 15 human fetal tissues; right, 15 human adult tissues), respectively. CircRNAs are most abundant in the human fetus brain compared with other tissues.

Circular RNAs has been shown to be tissue-specific. We compared the number of circRNAs detected in human tissues in the TSCD database^1^ and found that circRNAs are highly enriched in the brain (Figure 3; Xia et al., 2017). It was observed that the brain had a dominant role not only in the number of circRNAs but also in the frequency of circRNA hosting genes (approximately 20% of brain protein-coding genes produce circRNAs) (You et al., 2015). Another study reached a similar conclusion by comparing the human frontal cortex, thyroid gland, liver, and muscle (Rybak-Wolf et al., 2015). The enriched circRNAs are not uniformly distributed throughout the nervous system; it has been proven that they vary in different brain areas (Rybak-Wolf et al., 2015). A comparison of the circRNA expression of areas in the human and mouse brain showed that circRNAs were mostly enriched in the forebrain in mice and that the prefrontal cortex (PFC) had greater expression than the hippocampus (HC). Investigators assessed genome-wide expression of circRNAs in the HC and PFC of the mouse brain (Chen et al., 2018) and found an opposite result to that of Rybak-Wolf’s research; namely, circRNA expression in the HC was greater than that in the PFC. This finding may have occurred because Chen et al. (2018) chose data from the GEO database, while Rybak-Wolf et al. (2015) detected and analyzed these molecules on their own. Another reason may be the sample differences. However, both studies demonstrated the potential function of circRNAs in essential neuronal activities. Afterward, investigators further explored the exact enrichment localization of circRNAs in cells (You et al., 2015). Gene Ontology analysis indicated that circRNAs in the brain are mostly derived from several groups of genes related to synaptic function. Thus, high-resolution *in situ* hybridization (ISH) showed that localization of circRNAs was found in both the cell body and the dendrites of neurons (You et al., 2015). Furthermore, it was found that circRNAs were more abundant in synaptoneurosomes than whole-brain lysate and cytoplasm based on all expression cutoffs when they were normalized to host gene expression (Rybak-Wolf et al., 2015). The localization of circRNAs in the synaptic neuropil suggests that these molecules may play a role in the regulation of gene expression required for synaptic plasticity.

### Developmental-Stage-Specific Expression Profile

It has been proven that circRNAs are expressed in a developmental-stage-specific manner. During the maturation of primary neurons, most circRNAs (1,926 circRNAs) were found to be upregulated and only a few were downregulated (797 circRNAs) in the mouse brain (Rybak-Wolf et al., 2015). Investigation of *Drosophila* showed that the expression of circRNAs in neurons was increased throughout life (Westholm et al., 2014). During porcine embryonic brain development (E23, E42, E60, E80, E100, and E115) (Venø et al., 2015), circRNAs were increased from E23 to E60 and reached their peak at E60. Then, expression declined drastically with continuing reduction until E115. These implicit circRNAs may function at specific developmental periods and are important for neuronal function. In addition, investigators found that when compared with mRNA of their host gene, circRNAs were not expressed in the same way. The negative correlation between gene expression and the logarithm of the circular-to-linear ratio (CLR) indicates the independent function of circRNAs (Rybak-Wolf et al., 2015). The expression of circRNAs in the retina was also explored. It was found that many circRNAs were upregulated or downregulated during immature rat retinal development in P3, P7, and P12 (Han et al., 2017). Work from Chen et al. (2020c) in the mouse retina obtained a similar conclusion and indicated that the expression patterns of circRNAs differed from linear transcripts from the same host gene. The developmental stage-specific expression profile of circRNA suggests its important regulatory function in the development and differentiation of the nervous system. The data from the retina are not completely consistent with those from the brain; thus, further research is needed.

### Age-Related Accumulation

In addition to the early stages of development, investigators are also interested in the expression of circRNAs in the aging nervous system. To date, several studies have shown that age-related circRNA accumulation exists in various animals (Westholm et al., 2014; Gruner et al., 2016; Cortes-Lopez et al., 2018; Chen et al., 2019). *Drosophila melanogaster* RNA-Seq libraries reveal this phenomenon for the first time. Westholm et al. found that the expression of 262 circRNAs was significantly upregulated in 20-day heads versus 1-day heads (Westholm et al., 2014). The circRNAs identified in the cortex, HC and heart of 1- and 22-month-old mice were analyzed (Gruner et al., 2016). The study revealed that circRNAs showed significant upregulation in elderly cortex and hippocampal samples but not in the heart. Moreover, investigators performed additional validations using the cortex from an intermediate age of 6 months (Gruner et al., 2016). They found that half of the circRNAs were significantly increased between 1 and 6 months, and all detected circRNAs were significantly increased between 6 and 22 months. This result revealed that the age-dependent accumulation was progressive. In addition, aging-related accumulation of circRNAs was found in *C. elegans* and porcine brain (Cortes-Lopez et al., 2018; Chen et al., 2019). The latest research on rhesus monkeys showed that 11 circRNAs and host mRNAs are involved in regulating brain aging (Xu et al., 2020). One of them, circGRIA1, can regulate age-related synaptic plasticity by negatively regulating its host gene in the nucleus. Interestingly, the accumulation of circGRIA1 is also male-biased, which warrants further study.

The above results revealed that circRNAs are highly expressed in the nervous system. The uneven distribution of circRNAs in different brain regions indicates that they have potential functions in spatial learning and memory. The age-related accumulation of circRNAs suggests that they may contribute to neuronal aging and age-related diseases such as Alzheimer’s disease (AD) (Lukiw and Circular, 2013) and age-related macular degeneration (AMD) (Jin et al., 2019; Chen et al., 2020a). Current studies show some evidences in these diseases but still requires further research.

## circRNAs in Neurological Diseases

**FIGURE 4:**
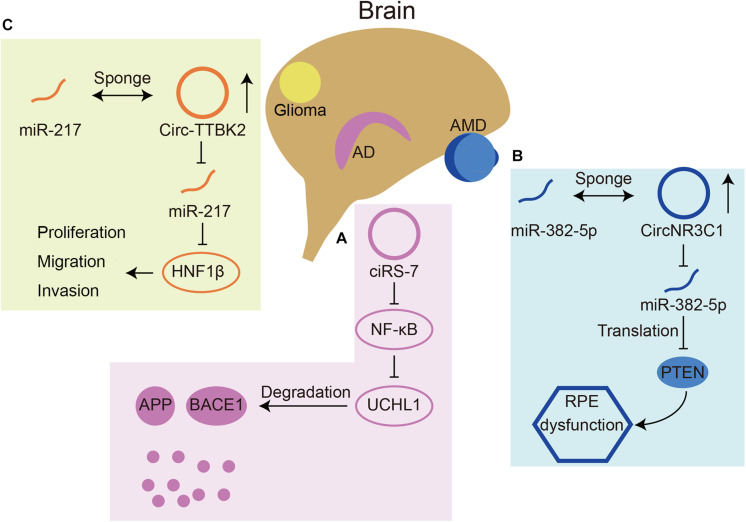
Three circRNAs that play roles in neurological diseases. **(A)** CiRS-7(also named circCDR1as) can inhibit translation of NF-κB, resulting in upregulated expression of UCHL1. UCHL1 can promote the degradation of APP and BACE1 in AD. **(B)** CircNR3C1 acts as a miR-382-5p sponge, which can inhibit translation of an AMD related gene PTEN. **(C)** Circ-TTBK2 acts as a miR-217 sponge, which can inhibit HNF1β. HNF1β can promote glioma malignancy.

With such abundant expression in the nervous system, circRNAs play important roles in neurological diseases, such as AD, Parkinson’s disease (PD), and immune-mediated demyelinating diseases (Ghosal et al., 2013; Lukiw and Circular, 2013; Zhao et al., 2016; He et al., 2019). Briefly, we summarized some circRNAs involved in neurological diseases (Table 1) and three representative ones were chosen for detailed depiction (Figure 4).

**TABLE 1:** CircRNAs in neurological diseases.

Disease/Model	Host		CircRNAs	Mechanism	Reference
AD		Human	circCDR1as	As miR-7 sponge	Zhao et al., 2016; Shi et al., 2017
		Human	circHOMER1	As miR-651 sponge	Dube et al., 2019
		Human	circCORO1C	As miR-105 sponge	Dube et al., 2019
AD	HN cell	Human	circHDAC9	Alleviated Aβ42-induced HN cell neurotoxicity via miR-142-5p	Zhang Y. et al., 2020
	cellular AD model	Rat	circ 0000950	circ 0000950 enhanced neuron apoptosis and inflammatory response in AD through acting as a miR-103 sponge	Yang et al., 2019

PD		Human	circCDR1as	As miR-7 sponge	Ghosal et al., 2013
		Mouse	circDLGAP4	miR-134-5p/CREB pathway	Feng et al., 2020

Immune-mediated demyelinating disease		Human	hsa circ 0087862	Biomarker	He et al., 2019
			hsa circ 0012077		

CNS injury	HT22 cells with oxygen-glucose deprivation/reoxygenation (OGD/R)	Mouse	mmu-circRNA-015947	mmu-miR-188-3p, mmu-miR-329-5p, mmu-miR-3057-3p, mmu-miR-5098, mmu-miR-683 sponge	Lin et al., 2016
	Microglia-induced hippocampal neuronal apoptosis	Rat	circPTK2	MiR-29b-SOCS-1-JAK2/STAT3-IL-1β pathway	Wang et al., 2019
	Traumatic injury	Rat	circ-Spidr	PI3K-Akt signaling pathway	Mao et al., 2019a
	Nerve crush model	Rat	circ-Ankib1	miR-423-5p, miR-485-5p, and miR-666-3p	Mao et al., 2019b

Retinal disease	RB	Human	hsa circ 0001649	AKT/mTOR signaling pathway	Xing et al., 2018
	AMD/RPE cell line	Human	circNR3C1	circNR3C1-miR-3 82-5p-PTEN network	Chen et al., 2020a
	Rat model of glaucoma	Rat	circ-ZRANB1	circ-ZRANB1/miR-217/RUNX2 network	Wang et al., 2018b
	Rat model of glaucoma	Rat	cZNF609	As miR-615 sponge	Wang et al., 2018a

Glioma	Glioma cell lines	Human	circ-ZNF264	As miR-4493 sponge	Zhang et al., 2019
		Human	circPCMTD1	As miR-224-5p sponge	Zheng et al., 2019
	Human tissue sample and cell line	Human	circ-TTBK2	As miR-217 sponge	Zheng et al., 2017
	Human brain sample	Human	circ-FBXW7	Encode protein	Yang et al., 2018
	Human tissue sample and cell line	Human	circPOSTN	CircPOSTN/miR-361-5p/TPX2 axis	Long et al., 2020

*AD, Alzheimer’s disease; PD, Parkinson’s disease; CNS, central nervous system; RB, retinoblastoma; AMD, Age-related macular degeneration.*

### AD

The miR-7 circRNA system was shown to be dysregulated in the hippocampal CA1 region of sporadic AD patients by Northern blot hybridization techniques and the circularity-sensitive circRNA probe RNase R (Lukiw and Circular, 2013). It has been demonstrated that ubiquitin protein ligase A (UBE2A) is a target downstream gene of miR-7. UBE2A is the core effector of the ubiquitin 26S proteasome system, which acts by proteolysis to remove amyloid peptides. The expression of miR-7 is significantly increased in the brains of sporadic AD patients, which may be related to the downregulation of circCdr1as expression (Zhao et al., 2016). In addition, circCdr1as can promote the degradation of APP and BACE1 via the proteasome and lysosome (Shi et al., 2017; Figure 4A). Furthermore, the atlas of cortical circular RNA expression in AD and normal patients’ brains showed that circRNA expression levels are significantly correlated with both neuropathological and clinical measures of AD severity (Dube et al., 2019). In this study, circHOMER1 was most significantly correlated with AD and may be involved in AD as a sponge of miR-651, which regulates the AD-related genes *PSEN1* and *PSEN2* (Agarwal et al., 2015). Recent *in vitro* studies found additional circRNAs involved in AD. In Aβ42-treated HN cells, circHDAC9 overexpression can promote cell viability and repress cell apoptosis and inflammation via sponging miR-142-5p (Zhang N. et al., 2020). In a rat cellular AD model, circ_0000950 was found to promote neuronal apoptosis and the inflammatory response in AD via sponging miR-103 (Yang et al., 2019). The mechanism of circRNAs in AD remains to be studied in the future, which will provide a new direction for the treatment of AD.

### PD

MiR-7 also plays a role in PD by regulating alpha-synuclein (Ghosal et al., 2013). Alpha-synuclein is overexpressed with the development of PD and plays an essential role in PD. Overexpression of miR-7 can reduce the level of alpha-synuclein (Doxakis, 2010). Considering the interaction between mir-7 and circCdr1as, circCdr1as may participate in the PD pathological process by acting as a sponge. In addition, investigators found that circDLGAP4 had neuroprotective effects by modulating the miR-134-5p/CREB pathway in a PD mouse model (Feng et al., 2020), although the mechanism underlying circRNAs in PD is still unclear.

### Immune-Mediated Demyelinating Disease

CircRNA expression was dysregulated in cerebrospinal fluid from patients with immune-mediated demyelinating disease compared with that of healthy controls (2,364 were upregulated and 2,730 were downregulated) (He et al., 2019). The enrichment analysis of GO and KEGG showed that these circRNAs are most likely to participate in the process of macromolecule metabolism, membrane-bound organelles and protein binding. CircRNAs can also influence the immune response of viral infections through binding with immune factors, for example, NF90 and NF110 (Li et al., 2017).

### CNS Injury

CircRNAs participate in various types of neuronal injury. To investigate the mechanisms of circRNAs in cerebral ischemia-reperfusion-injury (IRI)-induced neuron injury, Lin et al. (2016) tested circRNA expression in HT22 cells with oxygen–glucose deprivation/reoxygenation (OGD/R) and found that three circRNAs were upregulated and 12 were downregulated. It was further shown that circPTK2 could inhibit miR-29b expression. Downregulated miR-29b expression can upregulate the JAK2/STAT3 signaling pathway and finally lead to neuronal apoptosis induced by OGD-activated microglia (Wang et al., 2019). Irreversible axonal damage is the main cause of neurological dysfunction in neurodegenerative diseases or after traumatic injury. Mao et al. (2019a) found for the first time that circRNAs could be involved in axon regeneration of injured neurons. These results showed that circ-Spidr enhances axon regrowth *in vitro* and *in vivo*. In addition, the investigators found another circRNA that had the opposite effect. Circ-Ankib1 inhibits axon regeneration by inhibiting Schwann cell proliferation in the sciatic nerve after crush injury (Mao et al., 2019b). These studies indicated a therapeutic possibility of circRNAs for CNS injury.

### Retinal Disease

As the retina is part of the CNS, many retinal diseases have also been shown to be related to circRNAs. The expression of circ-ZRANB1 was significantly upregulated in the aqueous humor of a rat model of glaucoma. Circ-ZRANB1 is mainly derived from Müller cells, which can bind to miR-271 to regulate the expression of RUNX2. Finally, the circ-ZRANB1/miR-217/RUNX2 network influenced retinal neurodegeneration caused by Müller cells (Wang et al., 2018b). Circ-ZNF609 has a similar function in retinal neurodegeneration induced by glaucoma by binding with miR-615 (Wang et al., 2018a). In AMD patients, circNR3C1 expression was found to be downregulated in the blood serum (Chen et al., 2020a). Investigators further assessed the possible mechanism of this circRNA through RPE cell lines and found that circNR3C1 protected RPE functions via the circNR3C1-miR-382-5p-PTEN network (Figure 4B). Retinoblastoma (Rb) is an important cause of blindness in early childhood (Liu et al., 2020). CircRNAs have also been shown to be dysregulated in RB and some RB cell lines. It was found that hsa_circ_0001649 was significantly downregulated and could regulate apoptosis and cell proliferation by the AKT/mTOR signaling pathway (Xing et al., 2018). There are currently few studies that have assessed the mechanism of circRNAs in retinal diseases.

### Glioma

In addition, some studies have shown the function of circRNAs in nervous system tumors. Circ-ZNF264, circPCMTD1, and circ-TTBK2 (Figure 4C) promote cell proliferation, migration and invasion in glioma cell lines by regulating their miRNAs (Zheng et al., 2017, 2019; Zhang et al., 2019). Circ-FBXW7 has a tumor suppressor effect in glioma cells by encoding a novel protein and is positively correlated with the overall survival rate (Yang et al., 2018). A recent study found that, apart from regulating cell growth and apoptosis, circ POSTN participated in aerobic glycolysis in glioma via the miR-361-5p/TPX2 axis (Long et al., 2020). Additionally, a recent review reported that most circRNAs function as miRNA sponges in glioma (Sun et al., 2020).

CircRNAs also play a role in psychiatric diseases such as bipolar disorder and major depressive disorder (Luykx et al., 2019; Zhang Y. et al., 2020).

Overall, the aforementioned studies indicate that circRNAs play a key role in many neurological diseases. However, the underlying mechanisms of circRNAs in many diseases have not been fully elucidated. Further study is required to elucidate how circRNAs exert their effects biologically.

## Perspectives

CircRNAs are novel RNAs that are abundantly expressed in numerous organisms. Numerous studies have shown that circRNAs are enriched in the nervous system. Many of them are derived from genes related to synaptic function. Synapses are critical in information transmission and regulation of neuronal activities. CircRNAs are usually small, and many circRNAs can be detected in exosomes. They may be transported by synaptic vesicles to adjacent cells to transmit information. However, more evidence is required. A recent study by Xu et al. (2020) showed that circRNAs in the nucleus can regulate synaptic plasticity. This finding has encouraged more investigators to identify more mechanisms of circRNAs in the nervous system.

The rapid development of biochemical methods, such as BaseScope ISH and high-throughput circRNA sequencing analysis, provides investigators with powerful tools to investigate the exact subcellular location and interaction with other molecules. If we can determine the location of circRNAs in exact types of neurons, we may be able to distinguish more types of circRNAs and further elucidate the functions of the emerging RNAs. These studies will provide powerful guidance for clinical diagnosis and treatment.

The mechanism by which age-related circRNAs accumulate in the brain is still unclear, probably because of their circular structure. This characteristic makes them resistant to RNase R and difficult to degrade, ultimately leading to their age-related accumulation. However, recent studies have found sex-biased accumulation, indicating that this phenomenon may be caused by multiple factors.

## Author Contributions

M-LL and WW collected the data and drafted the manuscript. Z-BJ revised and approved the manuscript. All authors contributed to the article and approved the submitted version.

## Conflict of Interest

The authors declare that the research was conducted in the absence of any commercial or financial relationships that could be construed as a potential conflict of interest.

## Footnotes

1 http://gb.whu.edu.cn/TSCD
